# Single-cell–informed senescence programs underpin a machine-learning prognostic model robustly validated across melanoma cohorts

**DOI:** 10.3389/fcell.2026.1939452

**Published:** 2026-09-08

**Authors:** Su Peng, Jiaheng Xie, Xiaohu He

**Affiliations:** 1 Department of Plastic Surgery, The Affiliated Friendship Plastic Surgery Hospital of Nanjing Medical University, Nanjing, China; 2 Department of Plastic Surgery, Xiangya Hospital, Central South University, Changsha, China

**Keywords:** cellular senescence, gpi, machine learning, melanoma, prognostic signature, single-cell RNA-seq, tumor microenvironment, WGCNA

## Abstract

**Background:**

Cellular senescence (CS) shapes tumor evolution and the tumor microenvironment (TME), yet senescence-informed prognostic models for skin cutaneous melanoma (SKCM) remain limited. We aimed to develop and validate a robust senescence-related prognostic signature by integrating single-cell and bulk transcriptomic data with systematic machine-learning screening.

**Methods:**

Senescence activity was quantified in the scRNA-seq dataset GSE115978 using a CS-AUC score, followed by cell-type annotation and CS-AUC–associated gene prioritization. In TCGA-SKCM, weighted gene co-expression network analysis (WGCNA) identified senescence-related modules. Genes supported by both single-cell and bulk analyses were intersected to derive 100 candidate genes. Prognostic models were trained in TCGA and evaluated using C-index in six independent GEO cohorts (GSE19234, GSE22153, GSE53118, GSE54467, GSE59455, GSE65904) across 101 machine-learning strategies, selecting the best-performing algorithm. Survival, time-dependent ROC, and PCA were used for validation. Immune infiltration and TME associations were assessed by multi-algorithm deconvolution and ESTIMATE. Key model genes were further analyzed, and GPI was experimentally validated in A375 cells.

**Results:**

Single-cell analysis revealed marked heterogeneity of CS-AUC across cell types and identified CS-AUC–correlated genes. WGCNA in TCGA identified a senescence-associated red module, and intersection with the single-cell-derived senescence signals yielded 100 genes. Among 101 candidates, the GBM-based model achieved the best overall validation performance in GEO cohorts. The resulting riskScore significantly stratified overall survival across TCGA and all validation cohorts and showed stable predictive accuracy by time-dependent ROC. High-risk tumors exhibited an immune-depleted TME characterized by lower stromal/immune scores and higher tumor purity, along with altered cancer–immunity cycle activity. Within the GBM signature, GPI displayed the strongest positive correlation with riskScore, was upregulated in tumors, predicted worse survival, and was associated with metabolic/proliferative programs by GSEA. Functionally, GPI knockdown reduced A375 migration and clonogenic growth.

**Conclusion:**

We developed an integrative senescence-informed prognostic model for SKCM with strong external validation, immune/TME relevance, and experimental support. GPI emerges as a key risk-associated gene and potential therapeutic target within the senescence-related prognostic framework.

## Introduction

Skin cutaneous melanoma (SKCM) is one of the most aggressive forms of skin cancer and remains a major cause of skin cancer–related mortality worldwide (Abbas et al., 2014; Pavri et al., 2016; Klebanov et al., 2019). Despite remarkable progress in targeted therapy and immune checkpoint blockade, clinical outcomes are highly heterogeneous, ranging from durable long-term responses to rapid progression and early death (Mervic, 2012; Lens et al., 2009; Tas et al., 2011). This variability reflects profound inter- and intra-tumoral heterogeneity at genetic, transcriptional, metabolic, and microenvironmental levels (Zilberg et al., 2025). Therefore, developing robust and biologically interpretable prognostic models remains a priority, not only to improve risk stratification but also to provide insights into tumor evolution and immune escape mechanisms.

Cellular senescence represents a stable cell-state program characterized by irreversible growth arrest accompanied by extensive transcriptional reprogramming (Palmer et al., 2019). Senescent cells can accumulate during aging and in response to oncogenic stress, DNA damage, oxidative stress, and therapy exposure (Regulski, 2017). In cancer biology, senescence is increasingly recognized as a “double-edged sword” (Ou et al., 2021). On one hand, senescence can act as a tumor-suppressive barrier by restraining proliferation (Książek, 2022). On the other hand, senescent cells can secrete a complex senescence-associated secretory phenotype (SASP), which includes cytokines, chemokines, growth factors, and matrix-remodeling enzymes (Beck et al., 2020). SASP may remodel the tumor microenvironment (TME), drive inflammation, promote immune modulation, enhance metastatic potential, and contribute to therapy resistance (Birch and Gil, 2020). In melanoma, where immune surveillance and immune evasion are central determinants of patient outcome, senescence-linked programs may have particularly strong clinical relevance by coupling tumor-intrinsic stress responses to immune and stromal remodeling.

A key challenge in translating senescence biology to prognostic modeling is that senescence is not a uniform tumor property. Instead, senescence-related transcriptional features can arise in malignant cells and multiple non-malignant compartments, including fibroblasts, macrophages, endothelial cells, and T cells. Bulk transcriptomic analyses provide cohort-scale outcome associations but cannot resolve which cellular compartments drive senescence signatures or how senescence heterogeneity manifests across cell types (Xie et al., 2025). In contrast, single-cell RNA sequencing (scRNA-seq) enables cell-type–resolved characterization of senescence programs and their diversity, but it typically lacks large-scale longitudinal clinical endpoints and may be limited by sample size (Durante et al., 2020). Therefore, integrative approaches that combine the cell-state resolution of scRNA-seq with the clinical power of bulk cohorts are needed to identify senescence programs that are both biologically grounded and clinically predictive.

Here, we developed an integrative senescence-based prognostic framework for SKCM by combining single-cell and bulk transcriptomic evidence with comprehensive machine-learning screening. We first quantified cellular senescence activity in scRNA-seq (GSE115978) using an AUC-based senescence score and identified senescence-associated transcriptional programs across annotated cell types. We then performed weighted gene co-expression network analysis (WGCNA) in TCGA-SKCM to identify bulk senescence-related gene modules and intersected these genes with the single-cell senescence-supported features to derive a stringent candidate gene set. Next, we constructed prognostic models using 101 machine-learning strategies trained in TCGA and selected the optimal model based solely on performance across six independent GEO validation cohorts. We further evaluated survival stratification, time-dependent predictive accuracy, and risk-group separability, and investigated immune infiltration, TME metrics, and cancer–immunity cycle associations. Finally, we prioritized a key model gene (GPI) and conducted single-gene prognostic and pathway analyses, followed by *in vitro* functional validation in melanoma cells. Collectively, our study provides a robust senescence-informed prognostic model for SKCM with strong external generalizability, immunological relevance, and experimental support.

## Methods

### Data acquisition and preprocessing

Publicly available datasets were used in this study. The scRNA-seq dataset GSE115978 was obtained from the GEO database for single-cell analyses (Jerby-Arnon et al., 2018). Bulk RNA-seq and corresponding clinical annotations for TCGA-SKCM were downloaded and used as the training cohort. Independent GEO cohorts (GSE19234, GSE22153, GSE53118, GSE54467, GSE59455, and GSE65904) were collected as external validation datasets for prognostic modeling (Bogunovi et al., 2009; Jönsson et al., 2010; Mann et al., 2013; Jayawardana et al., 2015; Budden et al., 2016; Cabrita et al., 2020). For bulk expression matrices, gene identifiers were unified to official gene symbols; when multiple probes mapped to the same gene, the probe with the highest average expression was retained. Expression values were log2-transformed where appropriate (e.g., log2 (TPM+1) or log2-normalized microarray expression). Patients without survival time/status information were excluded from survival analyses.

### Single-cell RNA-seq processing and cell-type annotation

Single-cell analyses were performed using Seurat (v4.3). Cells were retained after quality control based on standard criteria (e.g., minimum detected genes per cell, removal of extremely high-feature cells, and mitochondrial gene percentage filtering). Cells were retained after quality control based on explicit criteria: retaining cells with detected genes between 200 and 6,000, total UMIs > 500, and a mitochondrial gene percentage threshold of < 15%. Data were normalized using Seurat’s log-normalization framework, followed by identification of highly variable genes. Scaled expression matrices were generated and principal component analysis (PCA) was performed. Graph-based clustering was conducted using the top principal components, and the cell embedding was visualized using t-SNE. Cell types were annotated based on canonical marker-gene expression and established lineage signatures (e.g., T cells, B cells, NK cells, macrophages, endothelial cells, CAFs, and malignant cells).

### Single-cell cellular senescence scoring (CS-AUC), stratification, and correlation analysis

A curated cellular senescence (CS) gene set was used to quantify senescence activity at the single-cell level. Per-cell CS activity was computed as an AUC-based gene set score (CS-AUC) using an AUC/ssGSEA-style scoring strategy. Cells were stratified into high-CS-AUC and low-CS-AUC groups using the median CS-AUC value as the cutoff. Differential expression between CS-AUC groups was performed using a Wilcoxon rank-sum framework (Seurat), and correlation between gene expression and CS-AUC was calculated (Spearman correlation). Senescence-associated genes were prioritized by combining (i) differential expression directionality (higher in high-CS-AUC) and (ii) positive correlation with CS-AUC, and the top-ranked genes were carried forward for bulk integration.

### Bulk senescence module discovery using WGCNA in TCGA

To identify co-expression modules associated with senescence in bulk tumors, WGCNA was performed using TCGA-SKCM expression data. A bulk-level senescence trait was computed for each TCGA sample using the same CS gene set (ssGSEA/AUC-style scoring). A soft-thresholding power was selected based on the scale-free topology criterion, and an adjacency matrix and topological overlap matrix (TOM) were constructed. Gene modules were identified using dynamic tree cutting and merged based on eigengene similarity. Module–trait relationships were evaluated by correlating module eigengenes with the bulk senescence trait, and the module showing the strongest association (red module) was selected. Module membership (MM) and gene significance (GS) were computed to quantify intramodular connectivity and trait relevance.

Integration of single-cell and bulk results to define a 100-gene candidate set.

Genes from the senescence-associated WGCNA module were intersected with single-cell senescence-supported genes (CS-AUC high/low differential signals plus correlation evidence). The overlap constituted the final 100-gene candidate set used for downstream machine-learning model construction.

### Prognostic model construction with 101 machine-learning strategies

Using the 100-gene candidate set, prognostic modeling was performed using a systematic screening framework comprising 101 machine-learning strategies, including single algorithms and hybrid pipelines combining feature selection and survival learners (e.g., stepwise Cox selection, LASSO/elastic net, Ridge, CoxBoost, random survival forest, supervised principal components, survival-SVM, partial least squares for Cox, and GBM-based survival modeling). All candidate models were trained in TCGA-SKCM. Model outputs were converted to a continuous riskScore for each patient using the corresponding prediction function of each algorithm.

### External validation and selection of the optimal model

Model performance was assessed only in external GEO cohorts (GSE19234, GSE22153, GSE53118, GSE54467, GSE59455, and GSE65904) using the concordance index (C-index). The final model was selected based on overall ranking across validation cohorts (e.g., mean/median C-index across GEO datasets). The GBM-based model was selected as the optimal model according to validation performance.

### Survival analysis, time-dependent ROC, and PCA visualization

Patients were dichotomized into high-risk and low-risk groups using the median riskScore within each cohort. Kaplan–Meier survival curves were compared using the log-rank test. Time-dependent ROC analysis was conducted to evaluate predictive accuracy at 1-, 3-, and 5-year time points. PCA was performed using scaled expression of the prognostic signature genes, and group separation was visualized in the first two principal components.

### Comparison with published PubMed prognostic signatures

Previously published SKCM prognostic signatures curated from PubMed were reproduced based on reported gene lists and scoring schemes (using published coefficients when available). For each cohort (TCGA and the GEO validation datasets), the C-index of each published model was computed and compared against the CSS/GBM-derived model under a consistent evaluation protocol.

### Immune infiltration, tumor microenvironment estimation, and cancer–immunity cycle analysis

Immune infiltration was quantified using multiple computational deconvolution approaches (TIMER, CIBERSORT, CIBERSORT-ABS, quanTIseq, MCPcounter, xCell, and EPIC) via an integrated pipeline. Tumor microenvironment (TME) scores (StromalScore, ImmuneScore, ESTIMATEScore, TumorPurity) were calculated using the ESTIMATE method. Correlations between riskScore and immune/TME metrics were computed using Spearman correlation, with multiple testing controlled using the Benjamini–Hochberg method. Cancer–immunity cycle activity (steps 1–7) was quantified using a cancer–immunity cycle scoring framework (TIP-like strategy), and associations with riskScore were evaluated by correlation analysis.

### Gene-level correlation profiling and single-gene analysis of GPI

Correlations between riskScore and each GBM-signature gene were evaluated using Spearman correlation. For *GPI*, tumor-versus-normal differential expression was tested in independent GEO datasets using the Wilcoxon rank-sum test. Prognostic relevance was evaluated using Cox proportional hazards regression across cohorts and endpoints where available, and Kaplan–Meier survival analyses were performed by *GPI*-high/low grouping (median cutoff). Functional associations of *GPI* were explored by GSEA using MSigDB Hallmark gene sets, and enrichment analyses were further extended to GO and KEGG collections using a ranked-gene strategy with multiple-testing adjustment.

### Cell lines and cell culture

Human melanoma cell lines A375 were purchased from the American Type Culture Collection (ATCC, Manassas, VA, United States) or the Cell Bank of the Chinese Academy of Sciences (Shanghai, China). Cells were maintained in high-glucose Dulbecco’s Modified Eagle Medium (DMEM; Gibco, Thermo Fisher Scientific, Waltham, MA, United States; Cat# 11965092) supplemented with 10% fetal bovine serum (FBS; Gibco, Cat# 10099141) and 1% penicillin-streptomycin solution (100 U/mL penicillin, 100 ug/mL streptomycin; HyClone, Logan, UT, United States; Cat# SV30010). All cells were cultured in a humidified incubator at 37 °C in an atmosphere containing 5% CO2.

### Lentiviral transduction and stable knockdown cell generation

To achieve stable knockdown of GPI, two independent short hairpin RNAs (shRNAs) targeting distinct regions of the human GPI transcript and a non-targeting scramble control shRNA (sh-NC) were designed and synthesized (GeneChem, Shanghai, China). The target sequences were summarized in Supplementary Table S1. The oligonucleotides were cloned into the lentiviral expression vector pLKO.1-puro. Recombinant lentiviruses were packaged in HEK-293T cells by co-transfecting the packaging plasmids psPAX2 and pMD2. G using Lipofectamine 3,000 (Invitrogen, Cat# L3000015). Viral supernatants were harvested at 48 h and 72 h post-transfection, filtered through 0.45-um PES membranes, and added to melanoma cells in the presence of eight ug/mL polybrene (Sigma-Aldrich, Cat# TR-1003). At 48 h post-transduction, stable pools were selected using two ug/mL puromycin (Solarbio, Beijing, China; Cat# P8230) for 7 days.

### RNA extraction and real-time quantitative PCR (RT-qPCR)

Total RNA was extracted from harvested cells using TRIzol reagent (Invitrogen, Cat# 15596026) following the manufacturer’s protocol. RNA concentration and purity were measured using a NanoDrop 2000 spectrophotometer (Thermo Fisher Scientific). Reverse transcription was performed using one ug of total RNA with the PrimeScript RT Reagent Kit with gDNA Eraser (Takara, Shiga, Japan; Cat# RR047A). Quantitative real-time PCR was performed on a CFX96 Real-Time PCR System (Bio-Rad, Hercules, CA, United States) using TB Green Premix Ex Taq II (Takara, Cat# RR820A). The cycling conditions were: 95 °C for 30 s, followed by 40 cycles of 95 °C for 5 s and 60 °C for 30 s. Relative expression levels of GPI were calculated using the 2^(-▲▲Ct) method, with GAPDH serving as the internal reference.

### *In vitro* scratch wound-healing assay

Melanoma cells (3 × 10^5 cells/well) were seeded into 6-well plates and grown until reaching 90%–95% confluence. A straight linear wound was generated across the cell monolayer using a sterile 200-uL pipette tip. Detached cells and cellular debris were removed by washing twice with phosphate-buffered saline (PBS). Cells were then incubated in serum-free DMEM (to eliminate confounding effects from cell proliferation) at 37 °C in 5% CO2. Wound margins were photographed at identical coordinate positions at 0 h and 24 h using an inverted phase-contrast microscope (Olympus IX73, Tokyo, Japan; ×40 magnification). Wound closure areas were quantitatively measured using ImageJ software. Migration rate was calculated as: Migration rate (%) = [(Wound Area at 0 h - Wound Area at 24 h)/Wound Area at 0 h] * 100%.

### Colony formation assay

Log-phase melanoma cells were trypsinized, counted, and seeded into 6-well plates at a density of 800–1,000 cells per well in complete growth medium (10% FBS). Plates were gently agitated to ensure uniform single-cell distribution and incubated at 37 °C with 5% CO2 for 10–14 days, with the culture medium refreshed every 3 days. When visible colonies appeared, cells were washed twice with PBS, fixed with 4% paraformaldehyde (PFA; Solarbio, Cat# P1110) for 20 min, and stained with 0.1% (w/v) crystal violet solution (Sigma-Aldrich, Cat# C0775) for 15 min at room temperature. Staining solution was rinsed off with distilled water and air-dried. Colonies containing more than 50 individual cells were manually counted under an inverted microscope and imaged. All assays were executed in triplicate.

### Statistical analysis

All statistical analyses were performed in R. Two-group comparisons used Wilcoxon or Student’s t-test as appropriate. Survival differences were assessed by log-rank test, and hazard ratios were estimated using Cox regression. Correlation analyses used Spearman correlation. Multiple comparisons were corrected using the Benjamini–Hochberg procedure, and p/q < 0.05 was considered statistically significant.

## Results

### Single-cell senescence scoring reveals heterogeneous CS-AUC patterns and senescence-associated genes in melanoma

**FIGURE 1:**
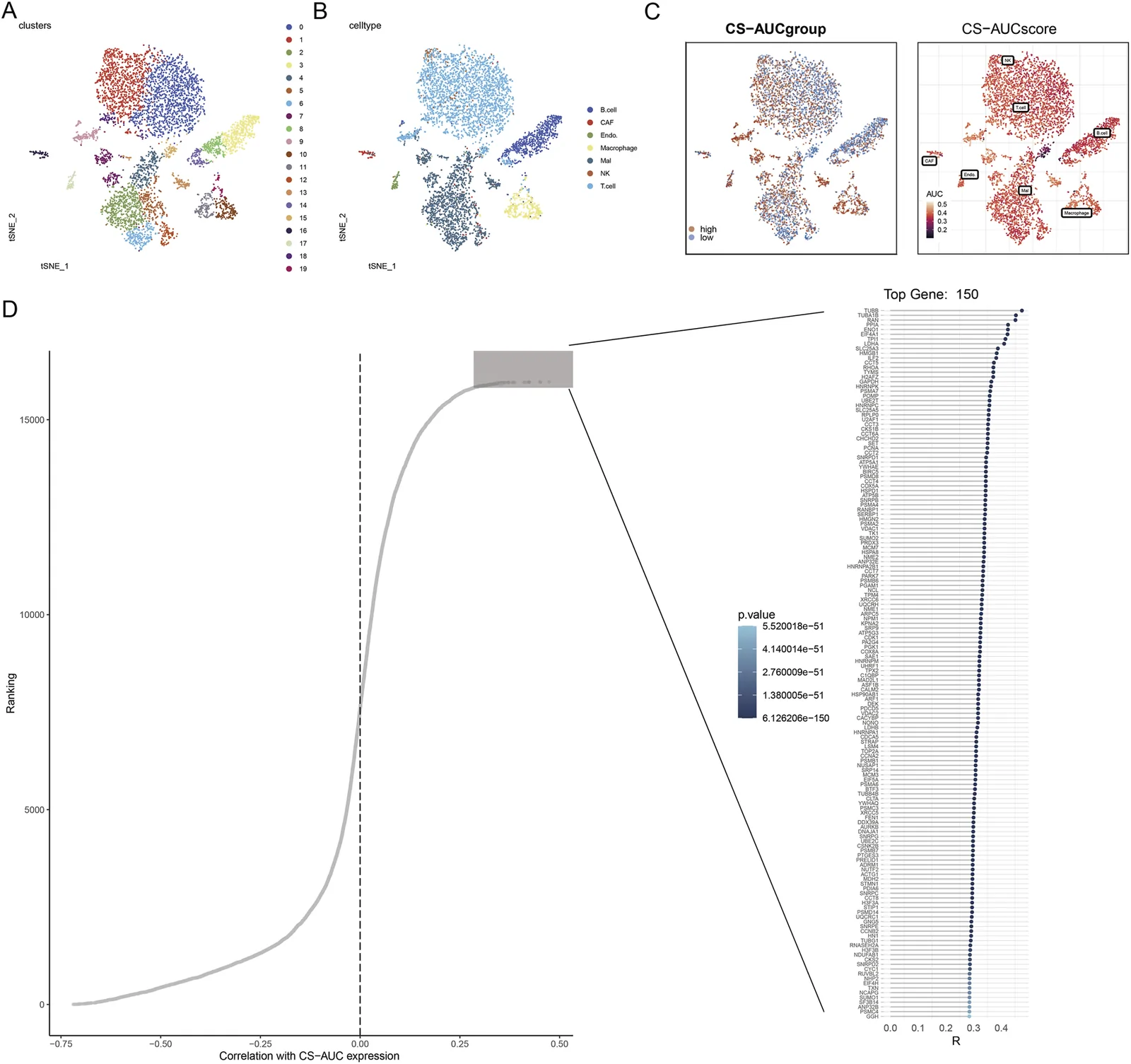
Single-cell analysis of GSE115978 defines cell populations and senescence heterogeneity. **(A)** t-SNE plot showing unsupervised clustering of scRNA-seq cells into 20 clusters. **(B)** t-SNE plot with cell-type annotation (e.g., malignant, T cells, B cells, NK cells, macrophages, CAFs, endothelial cells). **(C)** CS-AUC group (high vs. low) distribution on t-SNE (left) and CS-AUC score intensity and cell-type labeling (right). **(D)** Gene ranking by correlation with CS-AUC; the top 150 CS-AUC–associated genes are highlighted.

Using the scRNA-seq dataset GSE115978, we performed t-SNE–based unsupervised clustering and identified 20 transcriptionally distinct clusters (Figure 1A). Cell-type annotation resolved major populations including B cells, T cells, NK cells, macrophages, endothelial cells, CAFs, and malignant cells (Figure 1B). We next quantified cellular senescence activity using the CS-AUC score and stratified cells into high and low senescence groups, which showed broad but heterogeneous distribution across the t-SNE space and annotated cell compartments (Figure 1C). Correlation-based ranking further identified senescence-associated genes, with the top 150 genes exhibiting positive correlations with CS-AUC and extremely significant association signals (p-values reaching ∼10^−51^ to 10^−150^) (Figure 1D).

### WGCNA identifies a key senescence-related module in TCGA and yields 100 shared candidate genes with single-cell signals

**FIGURE 2:**
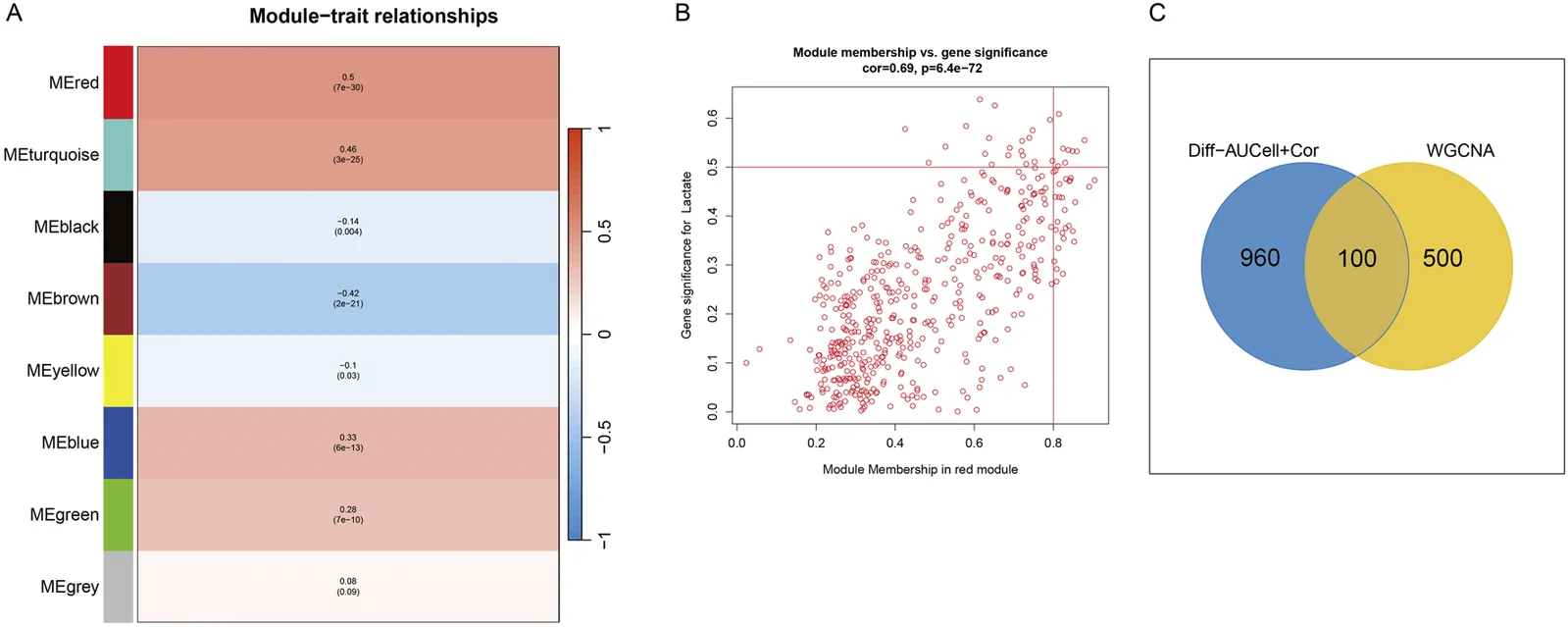
WGCNA in TCGA identifies a senescence-related red module and yields 100 intersected genes. **(A)** Scale-free topology fit index for soft-threshold selection. **(B)** Mean connectivity across soft-threshold powers. **(C)** Gene dendrogram and module color assignment before and after module merging. **(D)** Module–trait relationships identifying the red module as the most senescence-associated module. **(E)** Scatter plot of red-module membership versus gene significance. **(F)** Venn diagram showing intersection between WGCNA-derived genes and single-cell CS-AUC–supported genes, yielding 100 shared genes.

To convert single-cell insights into macroscopic patient cohorts, Weighted Gene Co-expression Network Analysis (WGCNA) was implemented on bulk transcriptomic data. Quantification of module-trait relationships revealed that the MEturquoise module possessed the most robust and significant positive correlation with the FS phenotype (Figure 2A). A highly significant correlation between module membership and gene significance for the trait was validated within this turquoise module (Figure 2B). Finally, by intersecting the genes from the MEturquoise module with the 86 candidate genes identified from single‐cell differential and correlation analyses (Diff‐AUCell+Cor), 42 overlapping core hub genes were successfully retrieved for model construction (Figure 2C).

### Machine-learning screening identifies GBM as the best-performing prognostic model across GEO validation cohorts

**FIGURE 3:**
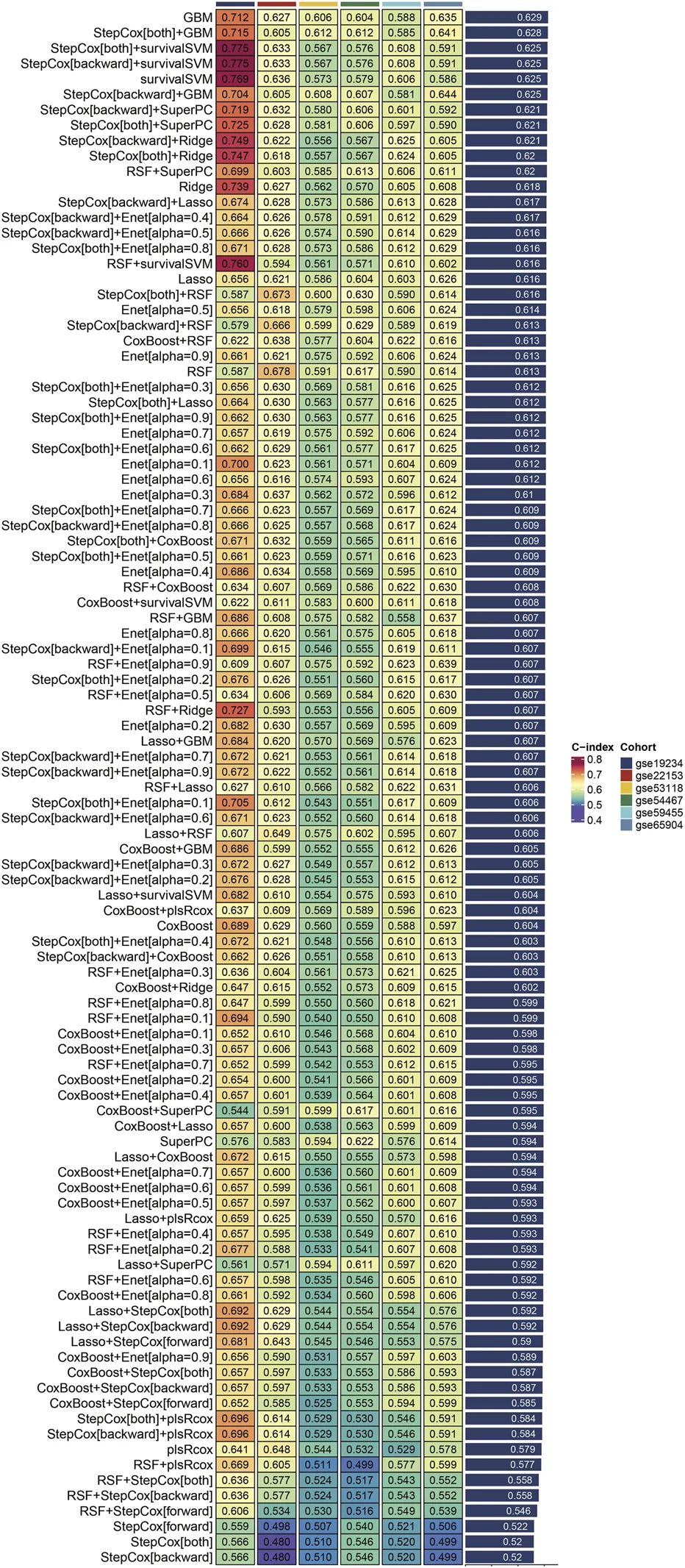
Systematic machine-learning benchmarking identifies GBM as the optimal prognostic model in external validation cohorts. Heatmap of C-index values across 101 machine-learning strategies evaluated in six GEO validation cohorts (GSE19234, GSE22153, GSE53118, GSE54467, GSE59455, GSE65904) after training in TCGA. The GBM-based model ranks best overall.

Using the 100 intersected genes, we constructed prognostic models with 101 machine-learning strategies, trained in TCGA and evaluated exclusively in external GEO validation cohorts (GSE19234, GSE22153, GSE53118, GSE54467, GSE59455, and GSE65904). Model performance was summarized by C-index across validation datasets (Figure 3). Among all candidates, the GBM-based model ranked first overall, achieving strong and consistent discrimination in GEO cohorts (C-index: 0.712 in GSE19234, 0.627 in GSE22153, 0.606 in GSE53118, 0.604 in GSE54467, 0.588 in GSE59455, and 0.635 in GSE65904; average 0.629) (Figure 3).

The GBM-derived risk score robustly stratifies survival and shows consistent time-dependent predictive accuracy.

**FIGURE 4:**
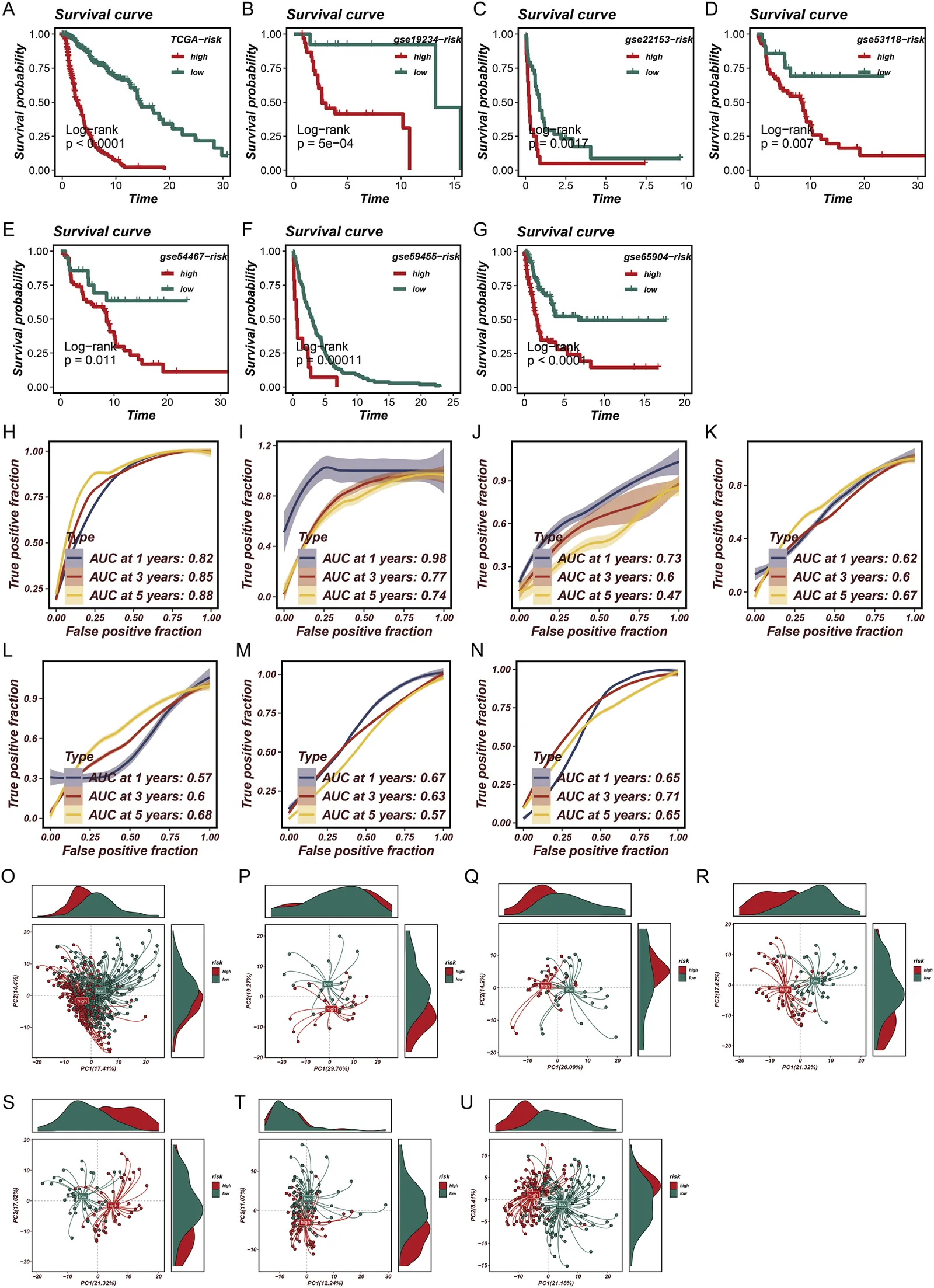
Prognostic performance of the riskScore across TCGA and GEO cohorts. **(A–G)** Kaplan–Meier overall survival curves comparing high- versus low-risk groups in TCGA and GEO cohorts from left to right: TCGA, GSE19234, GSE22153, GSE53118, GSE54467, GSE59455, and GSE65904. **(H–N)** Time-dependent ROC curves at 1, 3, and 5 years for the same cohorts (**(H)** TCGA; **(I)** GSE19234; **(J)** GSE22153; **(K)** GSE53118; **(L)** GSE54467; **(M)** GSE59455; **(N)** GSE65904). **(O–U)** PCA plots showing separation of high- and low-risk groups across cohorts (same order as above).

Based on the selected GBM model, patients were stratified into high- and low-risk groups. Kaplan–Meier analyses demonstrated significant survival separation in TCGA and all external GEO cohorts (TCGA p < 0.0001; GSE19234 p = 5e−04; GSE22153 p = 0.0017; GSE53118 p = 0.007; GSE54467 p = 0.011; GSE59455 p < 0.00011; GSE65904 p < 0.0001) (Figures 4A–G). Time-dependent ROC analyses further supported predictive performance, with AUCs (1/3/5-year) of 0.82/0.85/0.88 in TCGA, 0.98/0.77/0.74 in GSE19234, 0.73/0.60/0.47 in GSE22153, 0.62/0.60/0.67 in GSE53118, 0.57/0.60/0.68 in GSE54467, 0.67/0.63/0.57 in GSE59455, and 0.65/0.71/0.65 in GSE65904 (Figures 4H–N). PCA based on the signature also showed clear separation trends between risk groups across cohorts (Figures 4O–U).

### The CSS model shows competitive performance compared with previously published PubMed prognostic signatures

**FIGURE 5:**
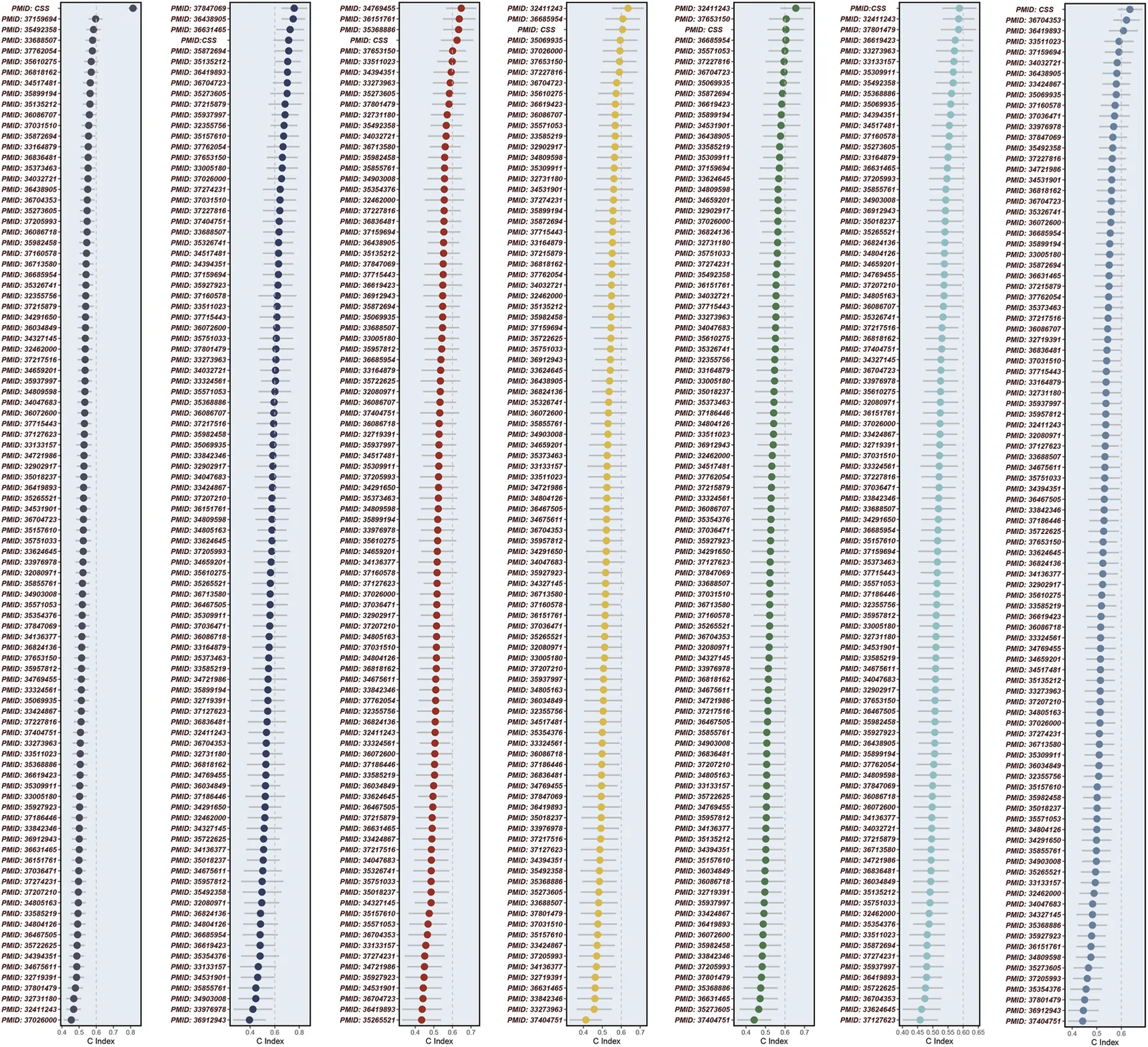
Comparison of the CSS prognostic model with published PubMed signatures across cohorts. Forest/dot plots comparing C-index performance of the CSS model and published prognostic signatures (labeled by PMID) across cohorts from left to right: TCGA, GSE19234, GSE22153, GSE53118, GSE54467, GSE59455, and GSE65904.

To benchmark clinical utility, we compared our CSS-based prognostic model against a large panel of previously published signatures curated from PubMed. Across cohorts shown from left to right (TCGA, GSE19234, GSE22153, GSE53118, GSE54467, GSE59455, and GSE65904), the CSS model consistently achieved high C-index performance, ranking favorably relative to most published models and demonstrating robust generalizability across independent datasets (Figure 5).

Forest/dot plots comparing C-index performance of the CSS model and published prognostic signatures (labeled by PMID) across cohorts from left to right: TCGA, GSE19234, GSE22153, GSE53118, GSE54467, GSE59455, and GSE65904.

### High-risk tumors exhibit an immune-cold microenvironment and suppressed cancer–immunity cycle activity

**FIGURE 6:**
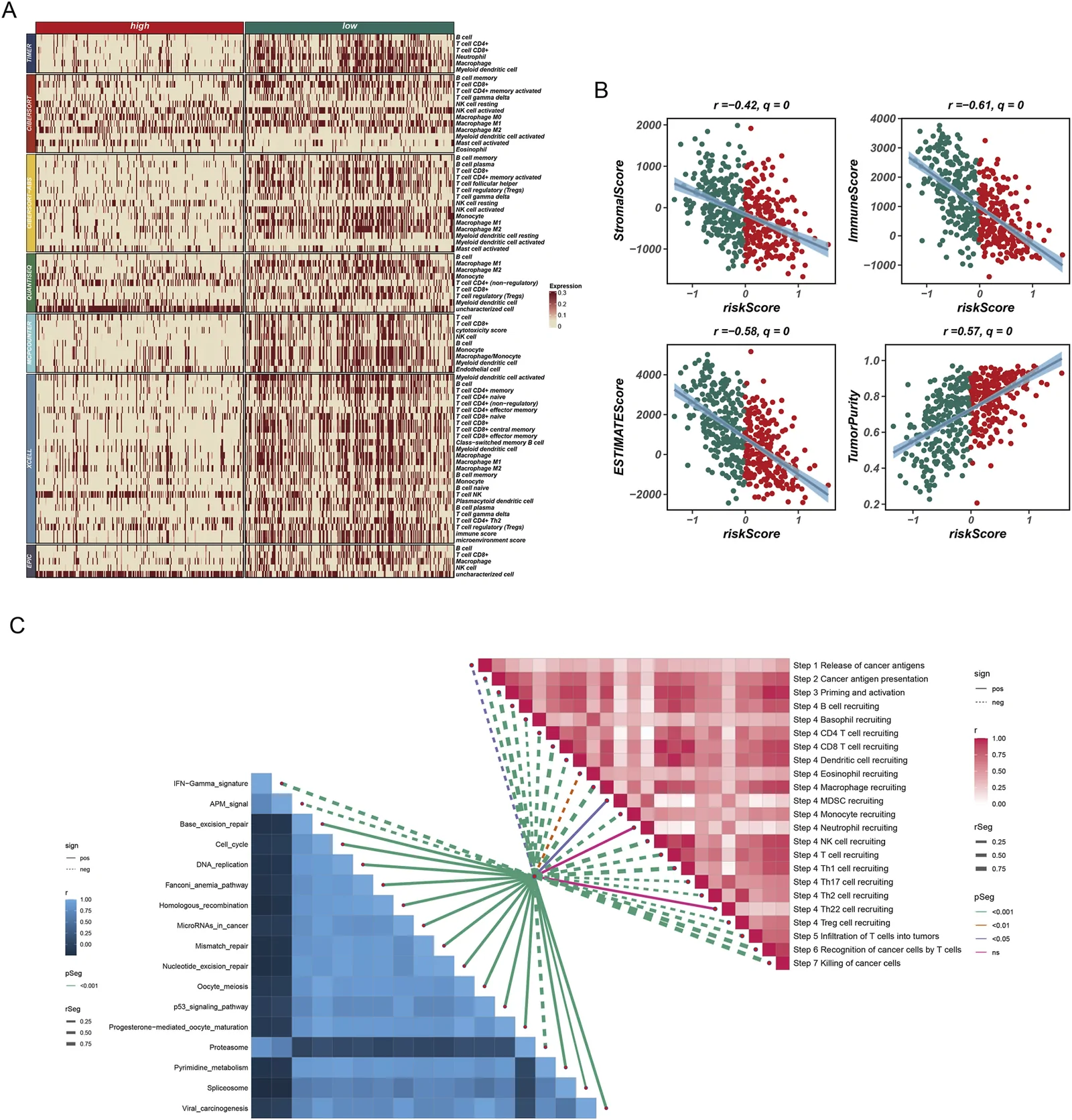
RiskScore is associated with immune infiltration, TME composition, and cancer–immunity cycle activity. **(A)** Heatmap summarizing immune cell infiltration estimates between high- and low-risk groups across multiple deconvolution algorithms (TIMER, CIBERSORT, CIBERSORT-ABS, quanTIseq, MCPcounter, xCell, EPIC). **(B)** Correlation of riskScore with StromalScore, ImmuneScore, ESTIMATEScore, and TumorPurity. **(C)** Correlation network/heatmap linking riskScore with pathway programs and cancer–immunity cycle steps.

Immune deconvolution across multiple algorithms (TIMER, CIBERSORT, CIBERSORT-ABS, quanTIseq, MCPcounter, xCell, and EPIC) revealed distinct immune infiltration patterns between risk groups (Figure 6A). ESTIMATE analysis showed that riskScore was negatively correlated with StromalScore (r = −0.42, q = 0), ImmuneScore (r = −0.61, q = 0), and ESTIMATEScore (r = −0.58, q = 0), while positively correlated with TumorPurity (r = 0.57, q = 0), indicating a comparatively immune-depleted, higher-purity tumor context in the high-risk group (Figure 6B). In addition, correlation analyses linking pathway programs and steps of the cancer–immunity cycle supported a broad association between higher riskScore and reduced immune activation/recruitment and effector processes, consistent with impaired antitumor immunity (Figure 6C).

### Risk score correlates with GBM signature genes, with GPI showing the strongest positive association

**FIGURE 7:**
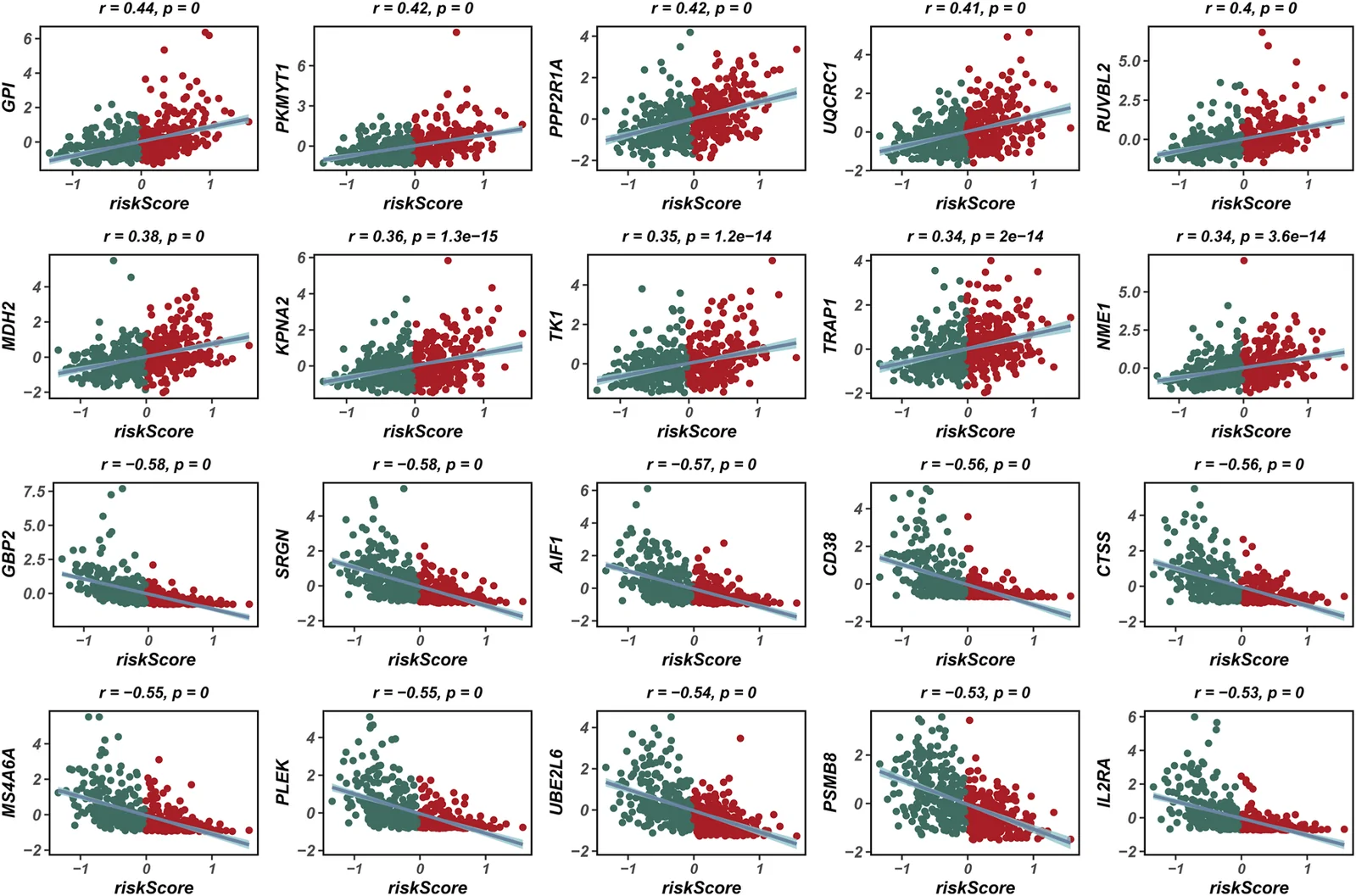
Correlation of GBM signature genes with riskScore identifies GPI as the top positively correlated gene. Scatter plots showing correlations between riskScore and representative GBM-signature genes, including positively correlated genes (e.g., GPI) and negatively correlated immune-associated genes, with correlation coefficients and p-values.

We next evaluated correlations between riskScore and expression of genes included in the GBM-derived signature. Multiple genes were significantly positively correlated with riskScore, led by *GPI* (r = 0.44, p = 0), followed by *PKMYT1* (r = 0.42), *PPP2R1A* (r = 0.42), *UQCRC1* (r = 0.41), *RUVBL2* (r = 0.40), and additional proliferation/mitochondrial-related genes (e.g., *MDH2, KPNA2, TK1, TRAP1, NME1*) (Figure 7). Conversely, several signature genes showed significant negative correlations with riskScore, including immune-related markers such as *GBP2* (r = −0.58), *SRGN* (r = −0.58), *AIF1* (r = −0.57), *CD38* (r = −0.56), *CTSS* (r = −0.56), *MS4A6A* (r = −0.55), *PLEK* (r = −0.55), *UBE2L6* (r = −0.54), *PSMB8* (r = −0.53), and *IL2RA* (r = −0.53), indicating that the GBM signature comprises both risk-enhancing and risk-protective components (Figure 7).

Scatter plots showing correlations between riskScore and representative GBM-signature genes, including positively correlated genes (e.g., *GPI*) and negatively correlated immune-associated genes, with correlation coefficients and p-values.

### Single-gene analyses highlight *GPI* overexpression, adverse prognosis, and enrichment of metabolic/proliferative programs

**FIGURE 8:**
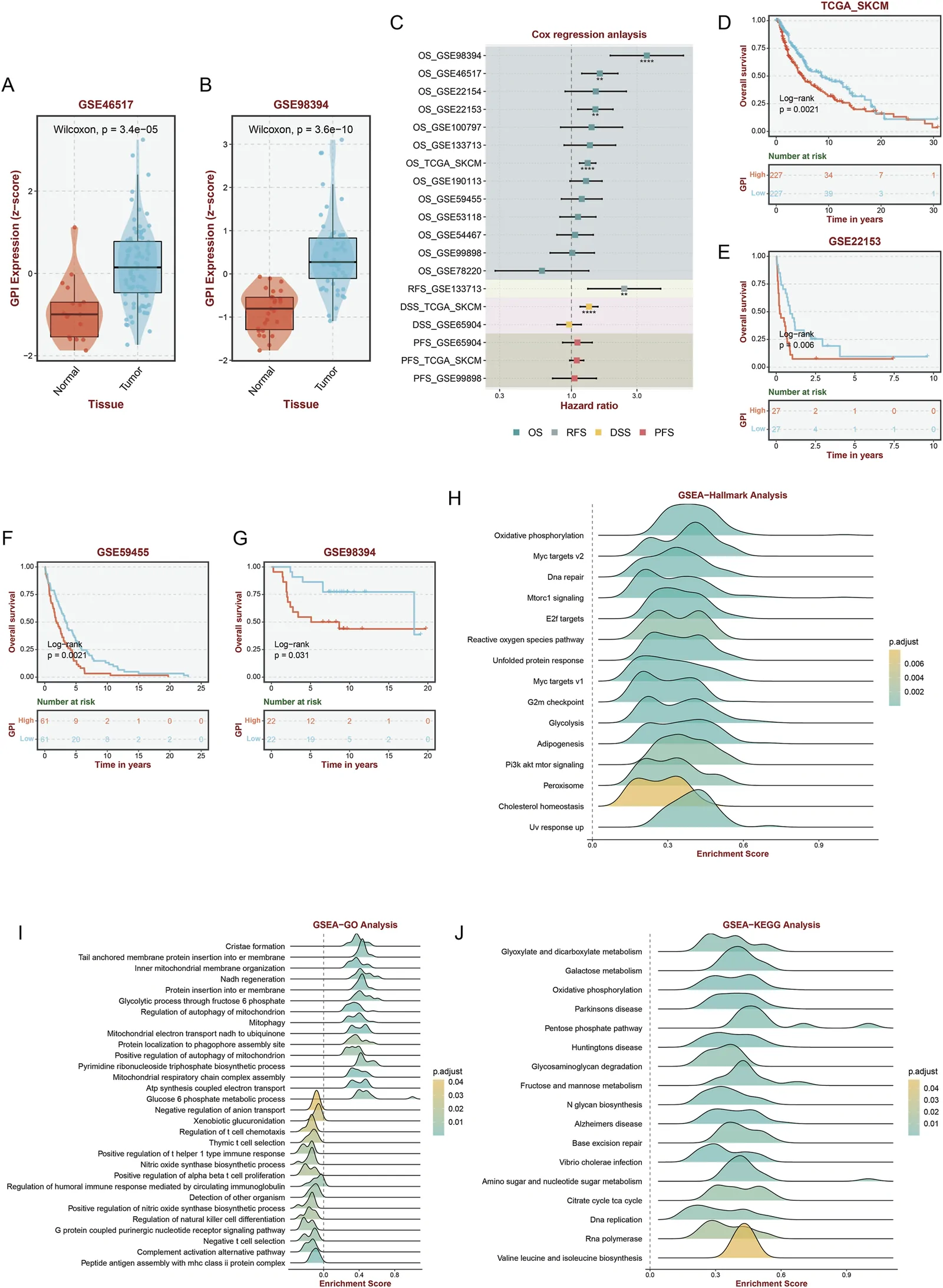
Single-gene analyses identify GPI as an overexpressed adverse prognostic marker with metabolic/proliferative enrichment. **(A,B)** GPI expression in tumor versus normal tissues in independent cohorts (GSE46517 and GSE98394). **(C)** Cox regression summary across cohorts/endpoints for GPI. **(D–G)** Kaplan–Meier overall survival stratified by GPI-high versus GPI-low in TCGA-SKCM, GSE22153, GSE59455, and GSE98394. **(H)** Hallmark GSEA comparing GPI-high versus GPI-low groups. **(I,J)** GO and KEGG GSEA enrichment results for GPI-associated programs.

Given that *GPI* showed the strongest positive correlation with riskScore, we performed single-gene validation. *GPI* was significantly upregulated in tumor versus normal samples in independent datasets (GSE46517 p = 3.4e−05; GSE98394 p = 3.6e−10) (Figures 8A,B). Pan-cohort Cox regression further supported its prognostic relevance across multiple survival endpoints (OS/RFS/DSS/PFS where available) (Figure 8C). Kaplan–Meier analyses consistently showed worse overall survival in the *GPI*-high group in TCGA-SKCM (p = 0.0021), GSE22153 (p = 0.006), GSE59455 (p = 0.0021), and GSE98394 (p = 0.031) (Figures 8D–G). Mechanistically, GSEA indicated that *GPI*-high tumors were enriched for hallmark programs linked to oxidative phosphorylation, MYC/E2F targets, DNA repair, mTORC1 signaling, G2M checkpoint, glycolysis, and PI3K–AKT–mTOR signaling (Figure 8H), with GO and KEGG enrichment further highlighting mitochondrial/energy metabolism and replication/repair-associated pathways (Figures 8I,J).

### GPI knockdown suppresses melanoma cell migration and clonogenic growth *in vitro*

**FIGURE 9:**
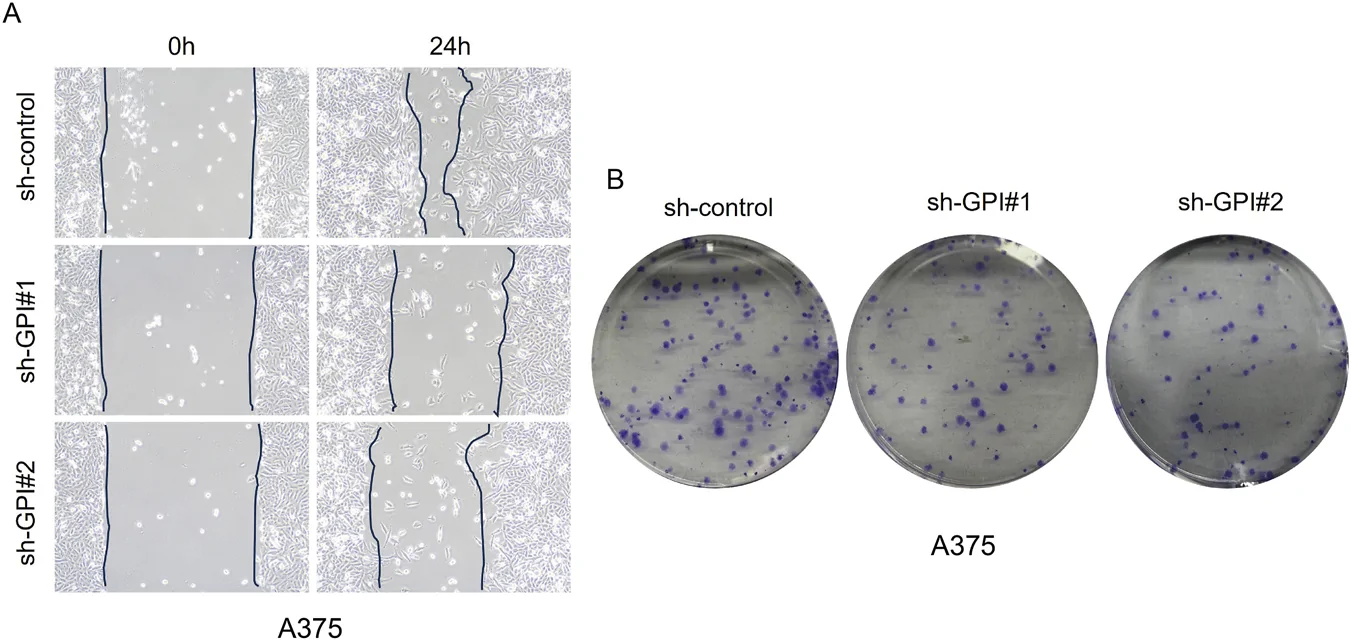
Cellular validation of GPI function in melanoma cells. **(A)** Wound-healing assay images at 0 h and 24 h showing reduced migration after GPI knockdown (sh-GPI#1/#2) compared with sh-control in A375 cells. **(B)** Colony formation assay showing decreased clonogenic growth in sh-GPI#1/#2 compared with sh-control in A375 cells.

To functionally validate the role of *GPI* in melanoma progression, we silenced *GPI* in A375 cells using two independent shRNAs (sh-*GPI*#1 and sh-*GPI*#2). The knockdown efficiency was verified by RT-qPCR, which confirmed significant downregulation of GPI mRNA levels in both sh-GPI groups compared with the scramble control (sh-NC) (Supplementary Figure S1). In the wound-healing assay, sh-control cells exhibited pronounced gap closure at 24 h, whereas both sh-*GPI* groups showed visibly delayed wound closure, indicating reduced migratory capacity upon *GPI* depletion (Figure 9A). Consistently, colony formation assays demonstrated that *GPI* knockdown markedly decreased the number and size of colonies compared with sh-control, suggesting that *GPI* is required for sustained proliferative and clonogenic potential in melanoma cells (Figure 9B).

## Discussion

In this study, we established a senescence-informed prognostic framework for SKCM by integrating single-cell senescence scoring, bulk co-expression network analysis, and large-scale machine-learning screening. Our approach addressed two common limitations of prognostic signature development: (i) inadequate resolution of senescence heterogeneity when relying solely on bulk transcriptomics, and (ii) model selection based on limited algorithmic exploration and insufficient external validation. By unifying single-cell and bulk evidence to derive a compact candidate set and benchmarking 101 learning strategies using independent GEO datasets, we identified a GBM-based model with strong generalizability.

A central observation is that senescence activity (CS-AUC) is heterogeneous across cell types and clusters in scRNA-seq, implying that senescence-related transcriptional programs cannot be inferred reliably from bulk expression alone. At single-cell resolution (GSE115978), GPI expression was found to be predominantly enriched in malignant cells and macrophages, indicating that its risk-enhancing role is closely anchored to both intrinsic cancer cell biology and specific microenvironmental dynamics. The downstream integration step—intersecting CS-AUC-supported genes with the TCGA-derived WGCNA red module—provided a stringent filter that likely enriched for genes that are both cell-state-relevant and preserved at the tissue level. This integrative design may partly explain the stability of performance across multiple validation cohorts.

Furthermore, considering melanoma’s molecular and anatomical diversity, cellular senescence signals represent a fundamental biological axis. While baseline senescence activity may vary across primary vs. metastatic lesions or driver mutation subtypes (e.g., BRAF or NRAS mutations), our model demonstrated robust stratifying power across heterogeneous external datasets that naturally encompass diverse primary/metastatic ratios and genomic backgrounds, underscoring its broad clinical applicability.

Clinically, the GBM-derived riskScore consistently stratified survival across TCGA and six independent GEO cohorts and showed time-dependent predictive accuracy in multiple datasets. Beyond prognostic discrimination, the riskScore also carried biologically interpretable signals: high-risk tumors exhibited lower immune and stromal infiltration scores and higher tumor purity, consistent with an “immune-cold” microenvironment. This phenotype was further supported by associations with cancer–immunity cycle steps, suggesting that risk stratification reflects not only tumor-intrinsic programs but also broader immune functional states. These findings are relevant for SKCM, where TME context and immune activity are tightly linked to clinical outcomes and therapeutic benefit.

Within the GBM signature, risk-associated genes showed bidirectional correlations with riskScore, indicating the model captures both risk-enhancing components (e.g., positively correlated metabolic/proliferative genes) and protective/immune-associated components (negatively correlated immune-related genes). Among positively correlated genes, *GPI* emerged as the top risk-associated marker, supported by tumor upregulation, adverse prognosis in multiple cohorts, pathway enrichment linked to metabolic and proliferative programs, and functional validation *in vitro*. The experimental data demonstrating reduced migration and clonogenicity after *GPI* silencing are consistent with a pro-tumorigenic role and provide mechanistic plausibility for its inclusion as a key component of the risk signature (Mishra et al., 2024).

Biologically, *GPI* (Glucose-6-phosphate isomerase) plays a well-established dual role: intracellularly, it functions as a crucial glycolytic enzyme fueling energy production and metabolic reprogramming, while extracellularly, it functions as autocrine motility factor (AMF) to stimulate cell migration and invasive signaling. Given that our GSEA highlighted marked enrichment of metabolic pathways alongside motility and proliferative programs, this dual intracellular/extracellular function aligns seamlessly with our observations, providing strong biological depth for its involvement in melanoma progression.

This study has limitations. First, most analyses are retrospective and rely on public cohorts with inherent technical differences (platform, batch effects, clinical heterogeneity). Although external validation across multiple GEO cohorts mitigates this concern, prospective clinical validation remains necessary. Second, the senescence scoring strategy depends on the selected senescence gene set; alternative senescence definitions may yield partially different gene programs. Third, while *GPI* was functionally validated, additional genes within the signature may also contribute to tumor progression and immune modulation, warranting deeper mechanistic investigation. Finally, the connection between senescence programs and therapy response—particularly immunotherapy—should be explored in future work using treatment-annotated cohorts (Deng et al., 2024).

Translating this machine-learning risk score into routine clinical practice presents both opportunities and hurdles (Zhao et al., 2022; Xie et al., 2023; Zeng et al., 2025). The primary challenge lies in standardizing multi-gene expression quantification across varying clinical diagnostic platforms (e.g., converting RNA-seq-derived models into multiplex RT-qPCR or targeted panel assays). Additionally, cross-platform batch effects, inter-laboratory technical variation, and establishing standardized risk score cutoffs for individual prospective patients represent crucial areas for future development before full clinical implementation (Zeng et al., 2025; Zeng et al., 2022).

In summary, we provide an integrative, externally validated senescence-informed prognostic model for SKCM and highlight *GPI* as a biologically and clinically relevant driver candidate. This work supports the utility of combining single-cell senescence resolution with bulk network biology and systematic machine-learning benchmarking to generate robust, interpretable prognostic tools.

## Data Availability

The original contributions presented in the study are included in the article/Supplementary Material, further inquiries can be directed to the corresponding author.

## References

[B1] Abbas O. Miller D. D. Bhawan J. (2014). Cutaneous malignant melanoma: update on diagnostic and prognostic biomarkers. Am. J. Dermatopathol. 36 (5), 363–379. 10.1097/DAD.0b013e31828a2ec5 24803061

[B2] Beck J. Turnquist C. Horikawa I. Harris C. (2020). Targeting cellular senescence in cancer and aging: roles of p53 and its isoforms. Carcinogenesis 41 (8), 1017–1029. 10.1093/carcin/bgaa071 32619002 PMC7422622

[B3] Birch J. Gil J. (2020). Senescence and the SASP: many therapeutic avenues. Genes Dev. 34 (23-24), 1565–1576. 10.1101/gad.343129.120 33262144 PMC7706700

[B4] Bogunovic D. O'Neill D. W. Belitskaya-Levy I. Vacic V. Yu Y. L. Adams S. (2009). Immune profile and mitotic index of metastatic melanoma lesions enhance clinical staging in predicting patient survival. Proc. Natl. Acad. Sci. U. S. A. 106 (48), 20429–20434. 10.1073/pnas.0905139106 19915147 PMC2787158

[B5] Budden T. Davey R. J. Vilain R. E. Ashton K. A. Braye S. G. Beveridge N. J. (2016). Repair of UVB-Induced DNA damage is reduced in melanoma due to low XPC and global genome repair. Oncotarget 7 (38), 60940–60953. 10.18632/oncotarget.10902 27487145 PMC5308628

[B6] Cabrita R. Lauss M. Sanna A. Donia M. Skaarup Larsen M. Mitra S. (2020). Tertiary lymphoid structures improve immunotherapy and survival in melanoma. Nature 577 (7791), 561–565. 10.1038/s41586-019-1914-8 31942071

[B7] Deng X. Liao T. Xie J. Kang D. He Y. Sun Y. (2024). The burgeoning importance of PIWI-interacting RNAs in cancer progression. Sci. China Life Sci. 67 (4), 653–662. 10.1007/s11427-023-2491-7 38198029

[B8] Durante M. A. Rodriguez D. A. Kurtenbach S. Kuznetsov J. N. Sanchez M. I. Decatur C. L. (2020). Single-cell analysis reveals new evolutionary complexity in uveal melanoma. Nat. Commun. 11 (1), 496. 10.1038/s41467-019-14256-1 31980621 PMC6981133

[B9] Jayawardana K. Schramm S. J. Haydu L. Thompson J. F. Scolyer R. A. Mann G. J. (2015). Determination of prognosis in metastatic melanoma through integration of clinico-pathologic, mutation, mRNA, microRNA, and protein information. Int. J. Cancer 136 (4), 863–874. 10.1002/ijc.29047 24975271

[B10] Jerby-Arnon L. Shah P. Cuoco M. S. Rodman C. Su M. J. Melms J. C. (2018). A cancer cell program promotes T cell exclusion and resistance to checkpoint blockade. Cell 175 (4), 984–997.e24. 10.1016/j.cell.2018.09.006 30388455 PMC6410377

[B11] Jönsson G. Busch C. Knappskog S. Geisler J. Miletic H. Ringnér M. (2010). Gene expression profiling-based identification of molecular subtypes in stage IV melanomas with different clinical outcome. Clin. Cancer Res. 16 (13), 3356–3367. 10.1158/1078-0432.CCR-09-2509 20460471

[B12] Klebanov N. Gunasekera N. S. Lin W. M. Hawryluk E. B. Miller D. M. Reddy B. Y. (2019). Clinical spectrum of cutaneous melanoma morphology. J. Am. Acad. Dermatol 80 (1), 178–188.e3. 10.1016/j.jaad.2018.08.028 30165162 PMC6492030

[B13] Książek K. (2022). Where does cellular senescence belong in the pathophysiology of ovarian cancer? Semin. Cancer Biol. 81, 14–23. 10.1016/j.semcancer.2020.11.021 33290845

[B14] Lens M. Bataille V. Krivokapic Z. (2009). Melanoma of the small intestine. Lancet Oncol. 10 (5), 516–521. 10.1016/S1470-2045(09)70036-1 19410196

[B15] Mann G. J. Pupo G. M. Campain A. E. Carter C. D. Schramm S. J. Pianova S. (2013). BRAF mutation, NRAS mutation, and the absence of an immune-related expressed gene profile predict poor outcome in patients with stage III melanoma. J. Invest. Dermatol. 133 (2), 509–517. 10.1038/jid.2012.283 22931913

[B16] Mervic L. (2012). Prognostic factors in patients with localized primary cutaneous melanoma. Acta Dermatovenerol Alp. Pannonica Adriat. 21 (2), 27–31. 23000937

[B17] Mishra A. K. Ye T. Banday S. Thakare R. P. Su C. T. T. Pham N. N. (2024). Targeting the GPI transamidase subunit GPAA1 abrogates the CD24 immune checkpoint in ovarian cancer. Cell Rep. 43 (4), 114041. 10.1016/j.celrep.2024.114041 38573857

[B18] Ou H. L. Hoffmann R. González-López C. Doherty G. J. Korkola J. E. Muñoz-Espín D. (2021). Cellular senescence in cancer: from mechanisms to detection. Mol. Oncol. 15 (10), 2634–2671. 10.1002/1878-0261.12807 32981205 PMC8486596

[B19] Palmer A. K. Gustafson B. Kirkland J. L. Smith U. (2019). Cellular senescence: at the nexus between ageing and diabetes. Diabetologia 62 (10), 1835–1841. 10.1007/s00125-019-4934-x 31451866 PMC6731336

[B20] Pavri S. N. Clune J. Ariyan S. Narayan D. (2016). Malignant melanoma: beyond the basics. Plast. Reconstr. Surg. 138 (2), 330e–340e. 10.1097/PRS.0000000000002367 27465194

[B21] Regulski M. J. (2017). Cellular senescence: what, why, and how. Wounds 29 (6), 168–174. 28682291

[B22] Tas F. Keskin S. Karadeniz A. Dağoğlu N. Sen F. Kilic L. (2011). Noncutaneous melanoma have distinct features from each other and cutaneous melanoma. Oncology 81 (5-6), 353–358. 10.1159/000334863 22248874

[B23] Xie J. Deng W. Deng X. Liang J. Y. Tang Y. Huang J. (2023). Single-cell histone chaperones patterns guide intercellular communication of tumor microenvironment that contribute to breast cancer metastases. Cancer Cell Int. 23 (1), 311. 10.1186/s12935-023-03166-4 38057779 PMC10702093

[B24] Xie J. Zhang P. Liu Y. Wu D. Ou X. Wang M. (2025). USP5-Mediated PD-L1 deubiquitination regulates immunotherapy efficacy in melanoma. J. Transl. Med. 23 (1), 778. 10.1186/s12967-025-06812-9 40640907 PMC12247207

[B25] Zeng C. Liu Y. He R. Lu X. Dai Y. Qi G. (2022). Identification and validation of a novel cellular senescence-related lncRNA prognostic signature for predicting immunotherapy response in stomach adenocarcinoma. Front. Genet. 13, 935056. 10.3389/fgene.2022.935056 36092903 PMC9453157

[B26] Zeng C. Wang J. Zhao S. Wei Y. Qi Y. Liu S. (2025). Multi-cohort validation of a lipid metabolism and ferroptosis-associated index for prognosis and immunotherapy response prediction in hormone receptor-positive breast cancer. Int. J. Biol. Sci. 21 (9), 3968–3992. 10.7150/ijbs.113213 40612672 PMC12223777

[B27] Zhao J. Li D. Xie S. Deng X. Wen X. Li J. (2022). Nomogram for predicting prognosis of patients with metastatic melanoma after immunotherapy: a Chinese population-based analysis. Front. Immunol. 13, 1083840. 10.3389/fimmu.2022.1083840 36618343 PMC9815596

[B28] Zilberg C. Ferguson A. L. Lyons J. G. Gupta R. Damian D. L. (2025). The tumor immune microenvironment in primary cutaneous melanoma. Arch. Dermatol Res. 317 (1), 273. 10.1007/s00403-024-03758-8 39825956 PMC11742903

